# Increased Extra‐Synaptic Localisation of the Presynaptic Protein, SV2A, in the Human Alzheimer's Disease Brain

**DOI:** 10.1002/alz70856_099018

**Published:** 2025-12-24

**Authors:** Stanley Williams, Samrah Siddiqi, Sulin Liu, Rutvi Patel, Leire Melgosa‐Ecenarro, Carola Radulescu, Samuel J Barnes, Paul M Matthews, Johanna S Jackson

**Affiliations:** ^1^ UK Dementia Research Institute at Imperial College, LONDON, London, United Kingdom

## Abstract

**Background:**

Synapse loss is a key component of Alzheimer's Disease (AD) and correlates with cognitive impairment. PET tracer, UCB‐J, binds to pre‐synaptic protein SV2a and is a potential biomarker of synapse density. SV2a is reportedly ubiquitously expressed at synapses but has been found in other locations making the origin of the PET signal difficult to interpret. Here, we determined the cellular and sub‐cellular distribution of SV2a.

**Method:**

Prefrontal and mid‐temporal gyral non‐diseased control (*n* = 14, Braak 0‐II) and AD (*n* = 14, Braak III‐VI) human *post‐mortem* tissue sections were stained with SV2a, synaptophysin, VGLUT1, VGAT, PSD‐95 and gephyrin to determine synaptic colocalisation. Further staining used MAP2, NfL, Iba1 and GFAP to map the distribution at non‐synaptic sites. Confocal imaging and Imaris were used for all analyses.

**Result:**

∼18% SV2a puncta colocalised with synaptophysin with no regional or disease‐state differences but a greater propensity to colocalise with VGLUT1 excitatory protein (∼11%) vs VGAT inhibitory protein (∼8%), similar to mouse models. The proportion of inhibitory and excitatory synapses with SV2a was ∼45% and 58% respectively. SV2a was found in axons (∼23%), and in somato‐dendritic compartments (∼7%) which was positively correlated with amyloid pathology. SV2a was observed in astrocytes and significantly increased in AD cortical layer 1 (∼24‐25%) vs controls (∼10‐16%) however only ∼2% was found in microglia throughout.

**Conclusion:**

We show that SV2a is not expressed at all synapses, with a substantial proportion found elsewhere in neurons and glia. Therefore, SV2a as a biomarker may not reveal the true extent of synapse loss in AD.

